# The InDeVal insertion/deletion evaluation tool: a program for finding target regions in DNA sequences and for aiding in sequence comparison 

**DOI:** 10.1186/1471-2105-5-173

**Published:** 2004-10-29

**Authors:** Sierra D Stoneberg Holt, Jason A Holt

**Affiliations:** 1 Department of Botany, Masaryk University, Brno, Czech Republic; 2 Rybkova 3, Brno, Czech Republic

## Abstract

**Background:**

The program InDeVal was originally developed to help researchers find known regions of insertion/deletion activity (with the exception of isolated single-base indels) in newly determined Poaceae *trn*L-F sequences and compare them with 533 previously determined sequences. It is supplied with input files designed for this purpose. More broadly, the program is applicable for finding specific target regions (referred to as "variable regions") in DNA sequence. A variable region is any specific sequence fragment of interest, such as an indel region, a codon or codons, or sequence coding for a particular RNA secondary structure.

**Results:**

InDeVal input is DNA sequence and a template file (sequence flanking each variable region). Additional files contain the variable regions and user-defined messages about the sequence found within them (e.g., taxa sharing each of the different indel patterns).

Variable regions are found by determining the position of flanking sequence (referred to as "conserved regions") using the LPAM (Length-Preserving Alignment Method) algorithm. This algorithm was designed for InDeVal and is described here for the first time.

InDeVal output is an interactive display of the analyzed sequence, broken into user-defined units. Once the user is satisfied with the organization of the display, the information can be exported to an annotated text file.

**Conclusions:**

InDeVal can find multiple variable regions simultaneously (28 indel regions in the Poaceae *trn*L-F files) and display user-selected messages specific to the sequence variants found. InDeVal output is designed to facilitate comparison between the analyzed sequence and previously evaluated sequence. The program's sensitivity to different levels of nucleotide and/or length variation in conserved regions can be adjusted. InDeVal is currently available for Windows in Additional file 1 or from .

## Background

Gaps caused by insertion and deletion events (indels) are often important features of DNA sequence data, which is widely used in phylogenetic studies [1-13]. Although some authors consider indels to be potentially misleading [1,14], others consider indels to be important characters [8,9,13,15,16] and have argued that treating them as missing data can weaken phylogenetic analyses [3,7,10,17]. Even though it is generally accepted that indels that cannot be unambiguously positioned make confident homology assessment impossible and, therefore, regions that contain them should be excluded from phylogenetic analyses [3,4,6,8], it has been proposed that even these regions are valuable if properly coded [15].

Phylogenetic estimation depends on accurate character homology assessment (sequence alignment) [4,8,18], which is made more difficult by the presence of indels [2-4,6,8,15]. Indel occurrence is context-dependent, and it has been repeatedly reported that indels tend to be found clustered into specific length-variable regions [1,3,5,6,8,19-21]. Accurate assessment of these regions (proper alignment and recognition of relative indel rate, reversals, parallel events, and multiple, overlapping events) is aided by comparison among many sequences from various taxonomic levels [4,8,19].

Sequence comparisons are complicated by the ambiguities gaps introduce into alignments. Finding a target region recognized from one alignment within another can be time consuming and difficult to perform accurately. Because of the limits of computer screen size and human analytical ability, alignments of hundreds of sequences are difficult to evaluate, even when they can be prepared.

Poaceae is one of the largest families of flowering plants and is economically important [22]. Lower-level phylogenies within the family often make use of the largely non-coding plastid sequence between the *trn*L (UAA) 5' exon and *trn*F (GAA), hereafter called *trn*L-F [17,23-28]. As of Apr 2, 2004, the NCBI Entrez Nucleotides database [29] contained 505 Poaceae *trn*L-F sequences. Comparing indel regions across these sequences can reveal patterns in indel behavior and aid researchers in creating accurate alignments. A discussion of the indel regions in these sequences is being prepared separately for publication.

The program InDeVal was originally developed to help researchers find known indel regions (with the exception of isolated single-base indels) in newly determined Poaceae *trn*L-F sequences and simultaneously compare them with 533 previously determined sequences (those mentioned above, plus 28 determined by SDSH). It is supplied with input files designed for this purpose. More broadly, the program is applicable for finding specific target regions (referred to as "variable regions") in DNA sequence. A variable region is any specific sequence fragment of interest, such as an indel region, a codon or codons, or sequence coding for a particular RNA secondary structure. The LPAM algorithm, which was specifically designed for InDeVal, is used to find sequence (referred to as "conserved regions") flanking the variable region in the analyzed sequence. InDeVal can find multiple variable regions simultaneously (28 indel regions in the Poaceae *trn*L-F files). The program's sensitivity to different levels of nucleotide and/or length variation in conserved regions can be adjusted.

## Implementation

### Input files

InDeVal uses three types of input files: one conserved region file, separate variable region files for each variable region, and a DNA sequence file (Table 1). The conserved region file contains a template of sequence immediately flanking the variable regions (regions of interest). A variable region file contains messages that indicate which permutation of the variable region is in the analyzed sequence. The sequence file contains a set of sequences to be analyzed with InDeVal. All files are plain text (ASCII). Conserved region and variable region files are in InDeVal-specific formats. Detailed instructions for creating them are in the help files. The sequence file is in FASTA-format.

A conserved region file contains at least one template, created by taking a representative sequence, removing the variable regions, and replacing them with variable region file names (Additional file 2). Multiple (15) templates were used in the Poaceae *trn*L-F sequences to accommodate single, large deletions that spanned otherwise conserved regions. Treating them the same as the other indels would have resulted in a few large, difficult-to-interpret variable regions. InDeVal performance is improved by designing templates with conserved regions at least 20 bases long on either side of each variable region. However, the program still functions using templates with only one conserved region, conserved regions only 4 bases long, and variable regions flanked by other variable regions (such as clearly distinguishable, adjacent indel regions varying at different rates, which are found in Poaceae *trn*L-F). Although InDeVal parameters can be adjusted to reflect different degrees of nucleotide and length variation in conserved regions, it is a good idea to use a representative sequence for template design, especially if some conserved regions are short. Additional templates can be designed to accommodate distantly related taxa. (In the Poaceae *trn*L-F files, separate templates were designed for Pooideae, Ehrhartoideae, and the PACCAD clade).

A variable region file contains the sequence variations the researcher expects in the region and some output information about each variation (Additional file 2). In the Poaceae *trn*L-F files, the output information is the list of taxa with each variation. For coding sequence, the output information could be the amino acids for which the variations code. If the user is interested only in knowing if a particular variation is present, the output could be simply "Yes". Variants not found in the variable region file are also reported by InDeVal. The program works if the variable region file is completely blank (variable regions are all bases found between template conserved regions), but it obviously cannot provide output messages in this case. Symbols can be used in variable region sequence to draw attention to specific features of interest. They are ignored during alignment, but are displayed in the Variable Region Sequence List Box (Additional file 2). The Poaceae *trn*L-F variable region files include spacing that emphasizes repeat motifs, hyphens to indicate that an entire variable region has been deleted, and stars to indicate possible inversion sites. InDeVal can help the user create these input files, comparing new sequences to those already included and indicating what adjustments should be made.

Sequence files are in a less stringent FASTA-format (Additional file 2) and can be in either orientation. They can include spaces, numbers, capital or small letters, IUPAC ambiguity symbols, and carriage returns without disrupting InDeVal function.

A conserved region file (TemplatePtrnLF.txt) and 28 variable region data files, designed from 533 Poaceae *trn*L-F sequences, are provided with InDeVal. These files are based on the first author's critical examination (to be published elsewhere) of various alignments of these sequences using the web-based programs BLAST 2 SEQUENCES [30] and/or CLUSTAL W multiple alignments [31].

### Aligning a sequence with a given template

**Figure 1:**
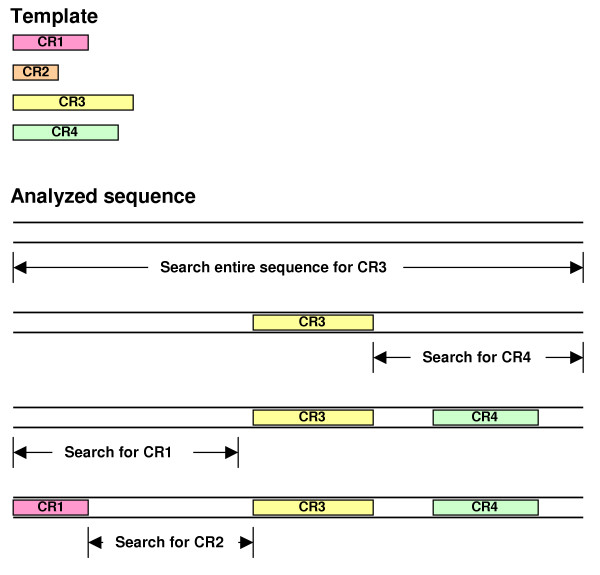
**Limiting search space with found conserved regions **This figure shows a schematic diagram of a template with four conserved regions of varying lengths (indicated by colored boxes and labeled CR1–CR4) and an analyzed sequence (indicated by two lines). Conserved regions are searched for in order of length, and each found conserved region limits the search space of future searches.

InDeVal begins by sorting a template's conserved regions by length. It then searches for each conserved region in the analyzed sequence (using LPAM – see below), proceeding from longest to shortest. Found conserved regions are used to limit the search space for future searches (Figure 1).

Sometimes conserved regions are not found or are found at multiple locations within their subsequence. The program records this information and proceeds to the next longest sequence. A conserved region that cannot be aligned is recorded as "not found." A conserved region with multiple possible alignments is recorded as "found", but none of its possible alignments is used to limit the searches for the remaining regions.

This template alignment algorithm preserves the ordering found in the template, giving priority to the alignment suggested by the longer conserved regions, which are assumed to be more reliable. Using the alignment of longer regions to reduce the search space for smaller regions minimizes the probability of finding ambiguous or incorrect alignments.

### Length-Preserving Alignment Method (LPAM)

InDeVal uses the LPAM algorithm, designed specifically for InDeVal and described here for the first time, to align the conserved regions within their subsequences. LPAM divides a conserved region into overlapping "words", strings of sequence of a user-defined length. That is, given a 4-base word length, the sequence "caatgt" would be represented as "caat", "aatg", "atgt". (Conserved regions shorter than the word length are found only if they match exactly.) LPAM searches the analyzed sequence for each of these words and notes multiple finds, single finds, and missing words. Each word is allowed one "vote" for a possible alignment, i.e., for the base in the analyzed sequence that begins the conserved region. If the word occurs once, it casts its vote for that alignment. If it occurs multiple times, its vote is divided equally among the possibilities. Words that are not found cast no vote. The alignment that receives the most votes is assumed to be the most probable.

LPAM permits a (user-definable) degree of length variation. A word's possible alignments are evaluated according to whether or not they agree with the most probable alignment for the region as a whole. Suppose, for example, that the most probable ungapped conserved region alignment would begin at base 53. If the tolerance is set to 3, LPAM will allow the first word to begin at any base from 50 to 56, preferring the possibility closest to 53. If the first word in the conserved region sequence has no acceptable alignment, LPAM searches for the first word that does have an acceptable alignment. All bases preceding this first found word are aligned with no gaps. In the above example, if the first and second words are not found and the third word aligns beginning at base 57, the first two bases of the conserved region will be aligned with bases 55 and 56.

Each subsequent base is aligned similarly. If the word it begins has no acceptable alignment, the base is aligned according to the previous acceptable word. If the word it begins has multiple acceptable alignments, the one closest to the previous alignment is chosen. Note that the range of acceptable alignments remains constant; it is a function of the initial voting and does not depend on any of the choices made in aligning individual bases.

The aligned bases from the analyzed sequence are compared with the template conserved region sequence, and a percentage similarity is established. If this is greater than the user-defined cut-off value, the conserved region is listed as found.

InDeVal then checks the most probable alignment that clearly differs from the first. (In the example above, this excludes any alignment beginning at bases 50–56.) If this alignment also yields a percentage similarity greater than the cut-off value, neither is selected, but the region is still considered to be "found" for purposes of measuring similarity to the template (see below).

The LPAM algorithm is able to align conserved regions despite both point mutations and indels. However, deletions in the first or last word of a conserved region are indistinguishable from point mutations (and are interpreted as such). LPAM proved effective in the Poaceae *trn*L-F sequences, where conserved regions are by definition length-conserved. In 533 sequences, there were only three instances where an indistinguishable mismatch was caused by an indel instead of a point mutation and resulted in misalignment. It is important to design templates so that the ends of conserved regions are not in sequence prone to indel activity.

The alignment suggested by LPAM is not necessarily locally maximal, i.e., there are cases where a slight adjustment of the alignment would produce a higher percentage of matched bases. Furthermore, a number of factors can prevent LPAM from finding a correct alignment. Repetitive sequence, resulting in multiple matches, can be very problematic. InDeVal may not be appropriate for analyzing repetitive sequence, and if it is used for this purpose, templates must be designed with great care. If every word contains a point mutation, the conserved region cannot be found. Indels can cause problems if they are dispersed so that several words are disrupted. In general, LPAM will correctly align regions if the mutations are clustered and the first and last base can be assigned correctly (which will be the case if they have no indels between them and the undisrupted word that will position them). Though hypothetical situations where LPAM would create incorrect alignments are easy to envision, in practice, the algorithm proved able to reliably determine where the conserved regions of a sequence begin and end. This is sufficient for the purposes of InDeVal. It is important to note that even when LPAM does not find a conserved region alignment, a possible alignment is displayed, and the user can rearrange it (to a limited extent) and interpret it.

### Choosing the best template

**Figure 2:**
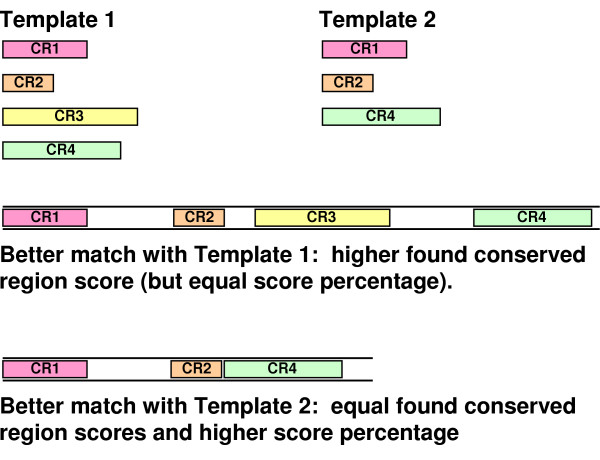
**Ranking templates **This figure shows a schematic diagram of two templates (conserved regions indicated by colored boxes) and two analyzed sequences (indicated by two lines). The first sequence is a better match with Template 1, because it has more conserved region bases in common. The second sequence is a better match with Template 2 (a major deletion template), because it matches a higher percentage of all the conserved regions in the template.

Once conserved regions have been found, InDeVal calculates a "found conserved region score" for each template. Each matched conserved region is given a value equal to the number of bases in that region in the template (regardless of how many individual bases actually matched), and these values are summed to give a score for the template. This prevents a template with a major deletion from being selected over a template with more matching conserved regions (as could happen if the deletion template were a better match at the base-per-base level). Templates with higher scores are ranked above those with lower scores (Figure 2).

If two templates have roughly equal found conserved region scores (to within a user-selected tolerance), the one with the higher "score percentage" (calculated by dividing the score by the total number of conserved region bases in the template) is ranked higher. This ensures that sequences that actually do have deletions will be matched with deletion templates, even though the templates (theoretically) have an equal number of bases in found conserved regions (Figure 2).

All the templates are ranked in this way and listed in the Template List Box in order. The highest ranked template is automatically selected, and its variable regions are analyzed. The user has the option of comparing the sequence to a template other than the one selected by InDeVal.

### Finding the correct variable region sequence

Each variable region is defined as the sequence between two specific conserved regions. If both flanking conserved regions are found, the bases between them are recorded, and the appropriate variable region file is searched for a matching string of bases.

Sequence between two found conserved regions is classified as a confused region if the template indicates that it should contain one or more conserved regions that were not found. Because the variable regions in a confused region are not clearly delimited, InDeVal searches the entire region for each variable region sequence in each applicable variable region file, and all potential matches are recorded. The user is able to select from among these possibilities, rearrange the display to reflect them, and study different sequence interpretations. In this manner, InDeVal is able to deal satisfactorily with most short conserved regions that cannot be found because of point mutations. In the Poaceae *trn*L-F file, one- and two-base conserved regions were incorporated into adjacent variable region files because they were impossible to find.

A short conserved region will be misaligned if a point mutation prevents it from being found in the proper place and a perfect match is found nearby. If this occurs, it is possible to set the program to disregard conserved regions of this size. (This situation has only been observed for 4- and 5-base conserved regions). The region can then be satisfactorily parsed using the confused region algorithm.

## Results and discussion

InDeVal has two output windows (Table 1, Additional file 2). The Sequence Analysis Window displays the analyzed sequence broken into conserved and variable regions and lists template information. From this window, the user can load template and sequence files, export the analysis to a text file, set LPAM parameters and warning message options, convert the sequence to its complement prior to analysis, and choose a template other than the one selected by InDeVal. For conserved regions, the template sequence is listed so that it can be compared directly with the analyzed sequence. For variable regions, if the analyzed sequence matches one of the variants in the variable region file, the message for that variant is displayed.

The Variable Region Analysis Window displays the variable region file name, variants proposed by InDeVal, and the sequence and length of the line currently selected in the Sequence Analysis Window (which can be any line from any sequence). The user can request displays of some or all of the sequences in the variable region file.

A text file can be created once the user is satisfied with the display in the Sequence Analysis Window. This file gives information about the sequence and template, lists the positions of the various conserved and variable regions, and shows the entire analyzed sequence. The user can choose whether or not variable region file information is displayed.

InDeVal for the Windows platform is available in Additional file 1. The source code for InDeVal in Microsoft Visual Basic 6.0 is available in Additional file 3. InDeVal analyzes only DNA sequences, but a version for protein sequences could be created using the source code. It would have to be altered to recognize amino acid sequence (all non-nucleotide letters are presently ignored), and an algorithm to recognize frame-shifts would be helpful.

## Conclusions

InDeVal is a program designed to search DNA sequence for target regions and to display information about them. It can find multiple target regions simultaneously and is relatively robust when challenged by conserved region variation and differences among analyzed sequences in length, spanned region, and format. InDeVal differs from other alignment software in that it breaks the analyzed sequence into user-defined units and emphasizes regions that are of most interest to the user. This makes it possible to quickly compare specific features among many hundreds of sequences. An advantage of InDeVal is that, while it can be used to quickly skim and classify hundreds of sequences, it displays all surrounding sequence. Therefore, if at any time questions arise about the initial classification, the researcher can recreate the InDeVal alignment, instantly find the area in question, and study it for alternative explanations.

## Availability and requirements

**Project name: **InDeVal

**Project home page: **

**Operating system(s): **Windows Platform

**Programming language: **Microsoft Visual Basic 6.0

**Other requirements: **None

**License: **None

**Any restrictions to use by non-academics: **No restrictions

## Authors' contributions

SDSH planned and oversaw the project, collected the data, prepared the data files, tested the program, and wrote the documentation. JAH suggested the project and designed, wrote, and debugged the program. Both authors read and approved the final manuscript.

## Supplementary Material

Additional File 1 **InDeVal 1.0 installation version **InDeVal is currently available for the Windows platform. Instructions for use can be found in the help files, which are included with the program and are accessible from the Sequence Analysis Window. An installation version of InDeVal 1.0 with accessory files can be obtained by clicking on the link below or by visiting . Running the installation program installs (in addition to system files) an InDeVal program directory that contains the Indeval.exe file, the 7 InDeValHelp.txt files, the InDeValOptions.txt file, the InDeValParams.txt (parameters) file, and a Templates subdirectory containing TemplatePtrnLF.txt and a Vr subdirectory with 28 Poaceae *trn*L-F variable region files. The program is archived using WinZip^® ^9.0. WinZip is available from . The zipped package has 3.2 MB. Click here for file

Additional File 2 **Annotated illustrations of InDeVal files and displays **This file (386 kB) is a 9-page pdf that contains annotated illustrations and explanations of InDeVal files and displays. The structure and format of the conserved region, variable region, sequence, and output files is illustrated. Annotated screenshots of the Sequence Analysis Window and Variable Region Analysis Window are also included. These illustrations, combined with the InDeVal help files, serve as the InDeVal Manual. Click here for file

Additional File 3 **InDeVal source code **The source code for InDeVal in Microsoft Visual Basic 6.0 can be obtained by clicking on the link below or by visiting . The files are archived using WinZip^® ^9.0. The package (61 kB) includes the 32 code files, the InDeVal icon and the bitmap from which it was constructed, and InDeValSourceCodeHelp.txt, a file with advice on orienting within the source code files. Click here for file

## Figures and Tables

**Table 1:** This table summarizes the inputs and outputs for InDeVal. It provides the names of the different InDeVal file types and displays, the number involved in analyzing a single portion of DNA, whether or not they are required for InDeVal function, their basic format, whether or not they are provided with the InDeVal package, when they are produced, and a brief description.

**Input**	Number	Required	Format	Provided	Description
Conserved region file	One	Yes	InDeVal-specific	Yes	Representative sequence with variable regions replaced by variable region file names
Variable region file	Multiple	No	InDeVal-specific	Yes (28)	List of sequences for which a variable region is to be searched and a message to be displayed for each
DNA sequence file	One	Yes	FASTA	No	Set of sequences to be analyzed

**Output**	Number	Produced	Format	Description	

Sequence Analysis Window	One per analyzed sequence	Always	Display	Displays analyzed sequence broken into user-defined units and allows InDeVal/user interaction	
Variable Region Analysis Window	One per variable region	Upon user-request	Display	Displays one variable region so that the sequence found in the analyzed sequence can be contrasted with those found in all other studied sequences	
Output file	One per analyzed sequence	Upon user-request	Similar to NCBI default display	Text file of the analyzed sequence, annotated with conserved and variable region positions and variable region sequence-specific messages	
