# Supplementary material for: The InDeVal insertion/deletion evaluation tool: a program for finding target regions in DNA sequences and for aiding in sequence comparison
Source: BMC Bioinformatics. 2004 Oct 29;5:173. doi: 10.1186/1471-2105-5-173 (PMC528782; doi:10.1186/1471-2105-5-173)
Supplement: Additional File 2 — Annotated illustrations of InDeVal files and displays This file (386 kB) is a 9-page pdf that contains annotated illustrations and explanations of InDeVal files and displays. The structure and format of the conserved region, variable region, sequence, and output files is illustrated. Annotated screenshots of the Sequence Analysis Window and Variable Region Analysis Window are also included. These illustrations, combined with the InDeVal help files, serve as the InDeVal Manual. [file 1471-2105-5-173-S2.pdf]

Conserved region file example: structure and format

```
# INDEVAL TEMPLATE FILE
# Sample Poaceae chloroplast partial trnL intron
# 3 Templates
```

Optional user bookkeeping information: # signals that the program should ignore this line.

```
>Pooideae Standard
1 91 gattgtattgagccttggtatggaaacctgctaagtggtaa
cagagaaaccctggaattaaaaaagggcaatcctgagcca
"VR\GreenfilePL-I.txt"
2 62 ctagaatacaaaggaaaaggataggtgcagagactcaatggaagctgttctaacgaatcgag
" VR\GreenfilePL-II.txt"
3 13 aattacgttgtgt
" VR\GreenfilePL-IIIa.txt"
" VR\GreenfilePL-IIIb.txt"
4 6 ttcctt
" VR\GreenfilePL-IIIc.txt"
5 5 taataa
" VR\GreenfilePL-IIId.txt"
6 7 gtatag
" VR\GreenfilePL-IIIe.txt"
7 17 gattaatcacagagccc
```

Template name: > is required; in provided file, Standard refers to a template without major deletions.

Conserved region: Numbers do not affect the program, but can be included by the user for bookkeeping purposes.

Variable regions: Must be given as exact file names in quotation marks; need not be separated by a conserved region.

Variable region path specification: Relative to directory containing conserved region file.

```
>Andropogoneae (intron)
1 91 gattgtattgagccttggtatggaaacctgctaagtg
cagagaaaccctggaatgaaaaatgggcaatcctgag
" VR\GreenfilePL-I.txt"
2 62 ctagaacccaaaggaaaaggataggtgcagag
" VR\GreenfilePL-II.txt"
3 23 aataac[ANDROPOGONEAE DELETION]ag
" VR\GreenfilePL-IIIe.txt"
4 17 gattaatcacagaaccc
```

Template name (Second template): In provided file, name refers to taxon for which this template's major deletion is specific; location of deletion given in parenthesis (not required by program).

Position and name of this template's major deletion: Square brackets are necessary to indicate that this is deletion information and not sequence.

```
>"Ehrharta" (IGS)
1 91 gattgtattgagccttggtatggaaacctgctaagtggcaac
cagagaaaccctggaattaaaaaggggcaatcctgagcca
" VR\GreenfilePL-I.txt"
2 62 ctagaacccaaaggaaaaggataggtgcagagactcaa
" VR\GreenfilePL-II.txt"
3 13 aattacgttgtgt
" VR\GreenfilePL-IIIa.txt"
" VR\GreenfilePL-IIIb.txt"
4 6 tccctt
" VR\GreenfilePL-IIIc.txt"
5 5 taatac
" VR\GreenfilePL-IIId.txt"
6 7 gtatag
" VR\GreenfilePL-IIIe.txt"
7 17 gattaatcacagaactc
```

Template name (Third template): In provided file, quotation marks indicate that most but not all sequenced members of this taxon contain this template's major deletion.

Numbers: Ignored by the program and used optionally to aid user orientation in the file; in provided file, the first figure numbers conserved regions consecutively, the second indicates the number of bases in a given conserved region.

Conserved region file example: structure and format

A conserved region file is organized into one or more templates (15 in the Poaceae *trn*L–F file, TemplatePtrnLF). The figure shows 3 annotated partial templates from TemplatePtrnLF. Each template begins with a name, written on a single line beginning with a greater than sign (>), and ends with the name of the next template. TemplatePtrnLF naming conventions, described in the annotations, are not required by InDeVal. Subsequent lines contain conserved region sequence and variable region file names. Characters between quotation marks (") are interpreted as variable region file names; all other characters are interpreted as conserved region sequence. The sequence can contain both upper and lower case letters. Characters not corresponding to a base are ignored except that information enclosed in brackets ([]) is displayed in the Sequence Analysis Window. Lines prefaced by a pound sign (#) are ignored completely and can be used to preface comments, which can aid orientation within the file.

Variable region file example: structure and format

gt gt >Chloridoideae<
gt >Phragmites, Panicoideae<
aa* >Nardus<
tt >Oryza, Aveneae, Arctagrostis, Castellia, Dactylis, Deschampsia, Dupontia, Festuca agustinii, F. burnatii, F. caerulescens, F. dimorpha, F. elviae, F. lasto, Poa, Puccinellia, Sesleria, Triticeae<
tc >Festuca dimorpha<
\- >Sclerochloa<
t >Festuca font-queri, F. mairei, Lolium, Schedonorus<

Species name occurs twice with different sequences

Species name indicates that members of that genus have more than one sequence variant

Higher taxon name indicates that all investigated members of that taxon shared the same sequence variant

Variable region sequence variants; Sequence motifs indicated with spacing; Star (*) indicates possible inversion site; Hyphen (-) indicates deletion of entire variable region

Names of taxa in which a given variant was found; Enclosed in greater than (>) and less than (<) signs

Note: InDeVal can function with no variable region file (though it will give error messages) or a blank variable region file. The messages (enclosed in >< signs) are strictly for the user's convenience.

This figure shows a hypothetical Poaceae *trn*L–F variable region file. A variable region file consists of a set of distinct sequence variations (highlighted in yellow in the figure), each followed by specific information (highlighted in blue), which is bracketed by the symbols > and <. The information (names of taxa in the Poaceae *trn*L–F files) will be displayed in the Taxa Boxes. Sequence can include spaces, numbers, and symbols (except >, <, and #) to highlight features of interest. These additional characters will not affect InDeVal analysis, but will be displayed in the Variable Region Sequence List Box.

The Poaceae *trn*L–F variable region file symbol conventions, described in the figure annotations and InDeVal help files, are not required by InDeVal. Higher-level taxa were assigned according to the classification in the NCBI Entrez Nucleotides database. Taxa below the species level were not used, because they were not uniformly applied within the database. The only hybrid taxon in the files is *Miscanthus* ×*giganteus* J. M. Greef & M. Deuter *ex* Hodkinson & Renvoize, because it was the only one with a unique indel pattern.

Sequence file example: structure and format

```
>gi|343400|gb|M13662.1|SRGCPTGLA Sorghum bicolor chloroplast split Leu-tRN
GAATTCAATGATTCAAAAAAAACTAAAAGATGGATTAAATTATACAAGGAATCCTGGTTTCAAAGAAAAG
TAAAATGGGGATATGGCGAAATCGGTAGACGCTACGGACTTGATTGTATTGAGCCTTGGTATGGAAACCT
GCTAAGTGGTAACTTCCAAATTCAGAGAAACCCTGGAATGAAAAATGGGCAATCCTGAGCCAAATCCACT
TTTTTCAAAAAAGTGGTTCTCAAACTAGAACCCAAAGGAAAAGGATAGGTGCAGAGACTCAATGGAAGCT

>NC_001320. Oryza sativa
4[illegible]561 gatatggcga aatcggtaga cgctacggac ttgattgtat tgagccttgg tatggaaacc
    46621 tgctaagtgg caacttccaa attcagagaa accctggaat taaaaaaggg gcaatcctga
    46681 gccaaatcca tgttttgaga aaacaagcgg ttctcgaact agaacccaaa ggaaaaggat
    46741 aggtgcagag actcaatgga agctgttcta acgaatcgag ttaattacgt tgtgttgcta

>Poa sibirica MC1, voucher specimen at MU, extracted 7/6 '02, df 1
a[illegible]gctacggacttgattgtattgagccttggtatggaaacctgctaagtggtaacttccaaattcagagaaaccc
tggaattaaaaaagggcaatcctgagccaaatccgtgttttgagaaaacaaggggttctcgaactagaatacaa
aggaaaaggataggtgcagagactcaatggaagctgttctaacgaatcgagttaattacgttgtgttgttagtgg
aattccttcgaaattctaaaaagaagggctttatacagctaataaacacgtatagatactgagatagcaaacgat
taatcacagagc
```

Required greater than (>) sign before sequence name.
Sequence name does not have a character limit, but can include only ONE carriage return.
Sequence name must be followed by a carriage return.

InDeVal sequence files use a less stringent FASTA-format. The sequences can include any pattern of capital or small letters, carriage returns, spaces, and numbers without disrupting InDeVal function. It is not necessary for the sequence to exactly match the region described in the template; it can begin or end before or after this region. If both ends of the sequence are within recognizable fragments of conserved regions, InDeVal will display only the template region spanned by the analyzed sequence (commonly the case for the *trn*L intron). However, if an end of the sequence is within an unrecognizable conserved region fragment or a variable region, the missing sequence will be displayed as blank lines. Although this does not affect InDeVal function, it can be distracting if the missing region is large (as is commonly the case for the *trn*L–F intergenic spacer). The researcher may find it more convenient to make an additional copy of the conserved region file under a different name and erase the unnecessary template lines from it.

Sample output file

```
# InDeVal Output File "gi344202.txt"
# Analyzed with template "Pooideae Standard"

NAME  gi|34420293|gb|AY177344.1| Festuca juncifolia chloroplast tRNA-Leu
      trnL) gene, intron
    (bases 1 to 534)

FEATURES
    1..77
        Conserved Region #1
    78..112
        Variable Region: "VR\GreenfilePL-I.txt"
        Festuca, Lolium, Schedonorus
    113..174
        Conserved Region #2
    175..176
        Variable Region: "VR\GreenfilePL-II.txt"
        Oryza, Aveneae, Nardus, Arctagrostis, Arctophila, Castellia
    177..189
        Conserved Region #3

ORIGIN
  1 TTGGTATGGA AACCTGCTAA GTGGTAACTT CCAAATTCAG AGAAACCCTG GAATTAAAAA
 61 AGGGCAATCC TGAGCCAAAT CCGTGTTTTG AGAAAACAAG GAGGTTCTCG AACTAGAATA
121 CAAAGGAAAA GGATAGGTGC AGAGACTCAA TGGAAGCTGT TCTAACGAAT CGAGTTAATT
181 ACGTTGTGT
```

File name, selected by user

Template, from Sequence Analysis Window

Base count, calculated

Sequence name, from Sequence File

Annotation, from Sequence Analysis Window; Bases in region, region type, Variable Region File name and (optional) data

Sequence, from Sequence File, formatted

This figure shows a hypothetical InDeVal output file. InDeVal can write the analysis from the Sequence Analysis Window to a text file. The text file includes information about the analysis, the analyzed sequence, and an annotation. The annotation shows which bases from the analyzed sequence were assigned to each of the separate regions. The user can decide whether or not to show information from the variable region files. This is not recommended with the Poaceae *trn*L–F files, because there are many variable regions and the information consists of very long taxa lists, which would only clutter the file. However, for other uses, it may be crucial that this information be included in the file.

Sequence Analysis Window screenshot

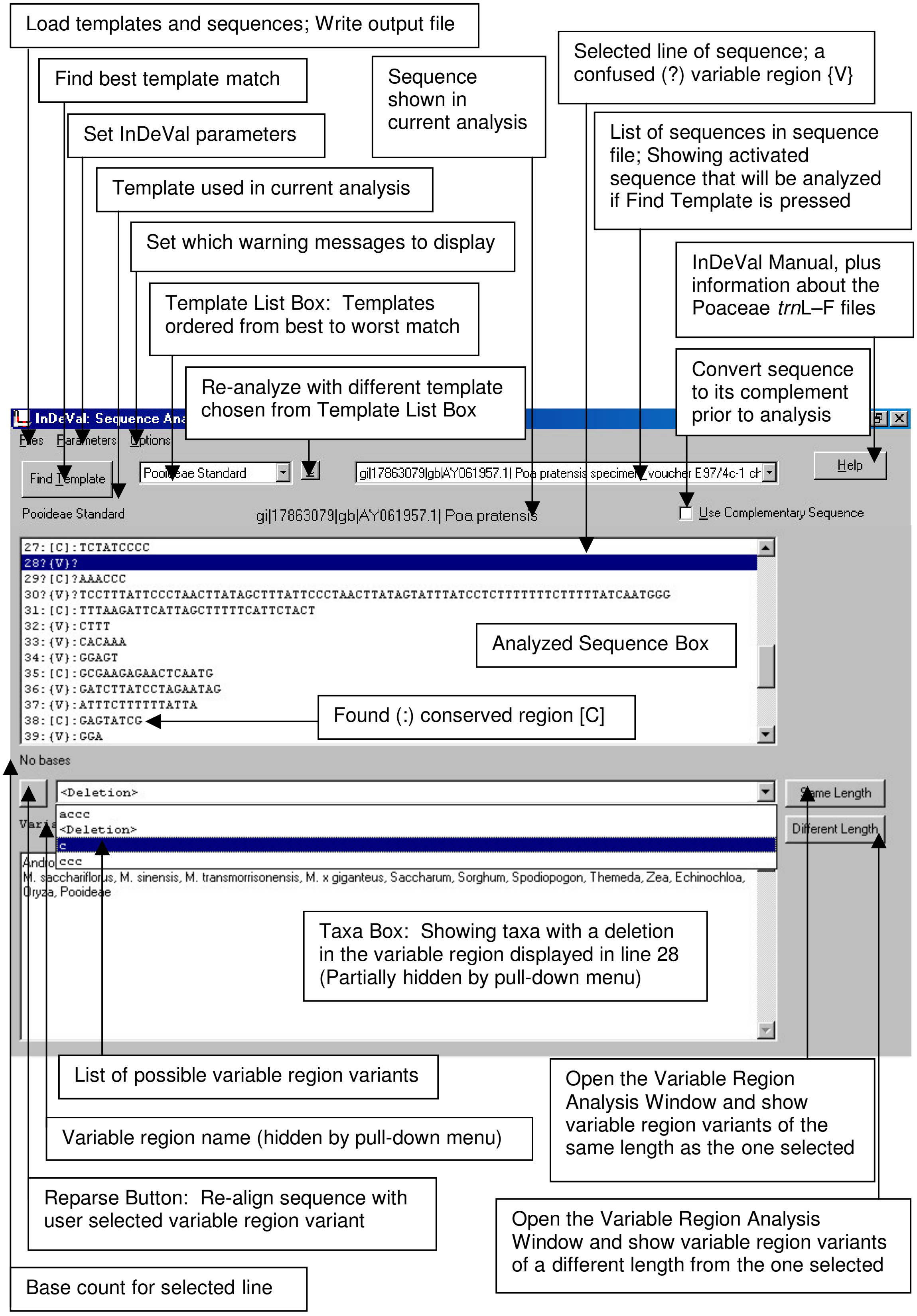

Sequence Analysis Window screenshot

From the illustrated window, the user loads template and sequence files and selects a sequence to analyze. By default, InDeVal processes the sequence using the template found to be the best match, but the user has the option of selecting one of the other templates and reprocessing the sequence with it. The processed sequence is displayed in the Analyzed Sequence Box as separate lines of conserved (C) and variable (V) regions. For found regions (those found by LPAM), the designation (C or V) is bracketed by colons (:), while for confused regions (those that had some ambiguity), the designation is bracketed by question marks (?). Selecting a line of sequence displays information about it: length, base count, whether it is a conserved or variable region, file name if it is a variable region, and the data file sequence. The analyzed sequence (in the Analyzed Sequence Box) can be directly compared with the sequence from the data files (displayed below). For a conserved region, the template name is displayed in the Taxa Box. For a variable region, the message text for the variation (names of the taxa that share it) appears in the Taxa Box. Buttons appear that lead to the Variable Region Analysis Window. If a confused variable region has more than one potential variant, a list of these is available. The button to the left of this list causes the surrounding confused regions to be rearranged so that the selected variant is displayed. This also works if there is only one variant that is not displayed correctly. Thus, the user can rearrange confused regions and study different interpretations of them. When the user is satisfied with the display, it is possible to export the information to an annotated text file.

Variable Region Analysis Window screenshot

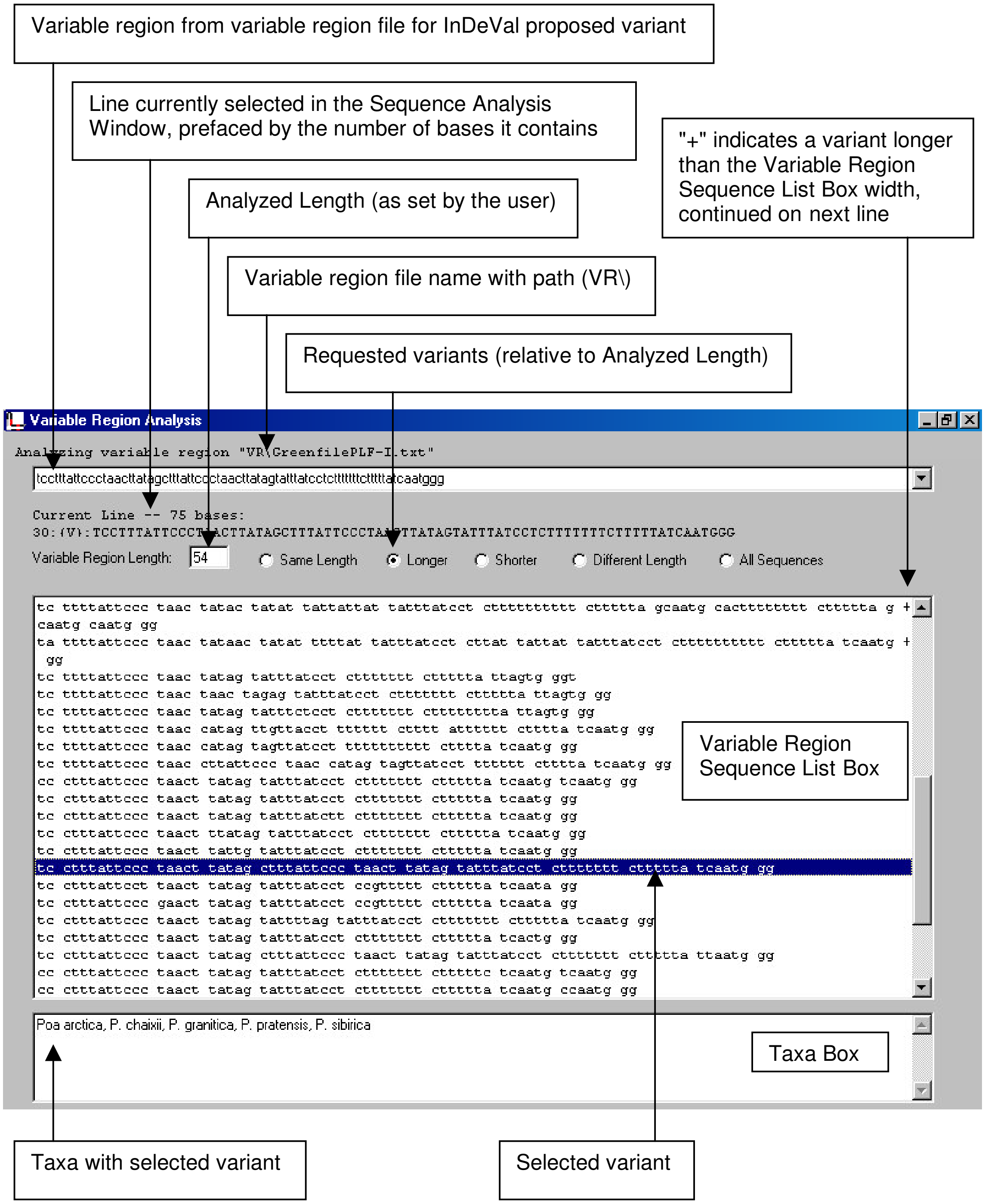

Variable Region Analysis Window screenshot

The Variable Region Analysis Window permits the user to explore and compare the different variable region sequence variants within a given variable region file. It is possible to display all the variants in a file. It is also possible to display variants that are the same length, different lengths, longer than or shorter than a chosen length. This chosen length is by default the length of the variant selected for the variable region by InDeVal, but can be set to any value. The Current Line Data refers to the line presently selected in the Sequence Analysis Window. This is very useful if InDeVal has for some reason selected an incorrect variant. For instance, a novel point mutation would mean that the actual variant was not in the variable region file. InDeVal would list the variable region as containing a deletion and list the variable region bases on the line below as a new insertion. By selecting this line in the Sequence Analysis Window and then re-activating the Variable Region Analysis Window, the user can compare the contents of the variable region file with those bases and discover the point mutation. Spaces are included in the Poaceae *trn*L–F variable region files to divide the sequence into readily interpretable motifs. If the InDeVal-suggested variant is present in the Variable Region Analysis Window, it is highlighted to alert the user to its presence.
