# Supplementary figures and images for: The InDeVal insertion/deletion evaluation tool: a program for finding target regions in DNA sequences and for aiding in sequence comparison
Source: BMC Bioinformatics. 2004 Oct 29;5:173. doi: 10.1186/1471-2105-5-173 (PMC528782; doi:10.1186/1471-2105-5-173)

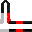

Supplement: Additional File 3 — InDeVal source code The source code for InDeVal in Microsoft Visual Basic 6.0 can be obtained by clicking on the link below or by visiting . The files are archived using WinZip® 9.0. The package (61 kB) includes the 32 code files, the InDeVal icon and the bitmap from which it was constructed, and InDeValSourceCodeHelp.txt, a file with advice on orienting within the source code files. [file 1471-2105-5-173-S3.zip › InDeVal.bmp]
